# Ebolavirus in West Africa, and the use of experimental therapies or vaccines

**DOI:** 10.1186/s12915-014-0080-6

**Published:** 2014-09-26

**Authors:** Thomas Hoenen, Heinz Feldmann

**Affiliations:** Laboratory of Virology, Division of Intramural Research, NIAID, NIH, 903 S 4th St, Hamilton, MT 59840 USA

## Abstract

Response to the current ebolavirus outbreak based on traditional control measures has so far been insufficient to prevent the virus from spreading rapidly. This has led to urgent discussions on the use of experimental therapies and vaccines untested in humans and existing in limited quantities, raising political, strategic, technical and ethical questions.

## Ebolavirus outbreaks and disease

**Figure 1:**
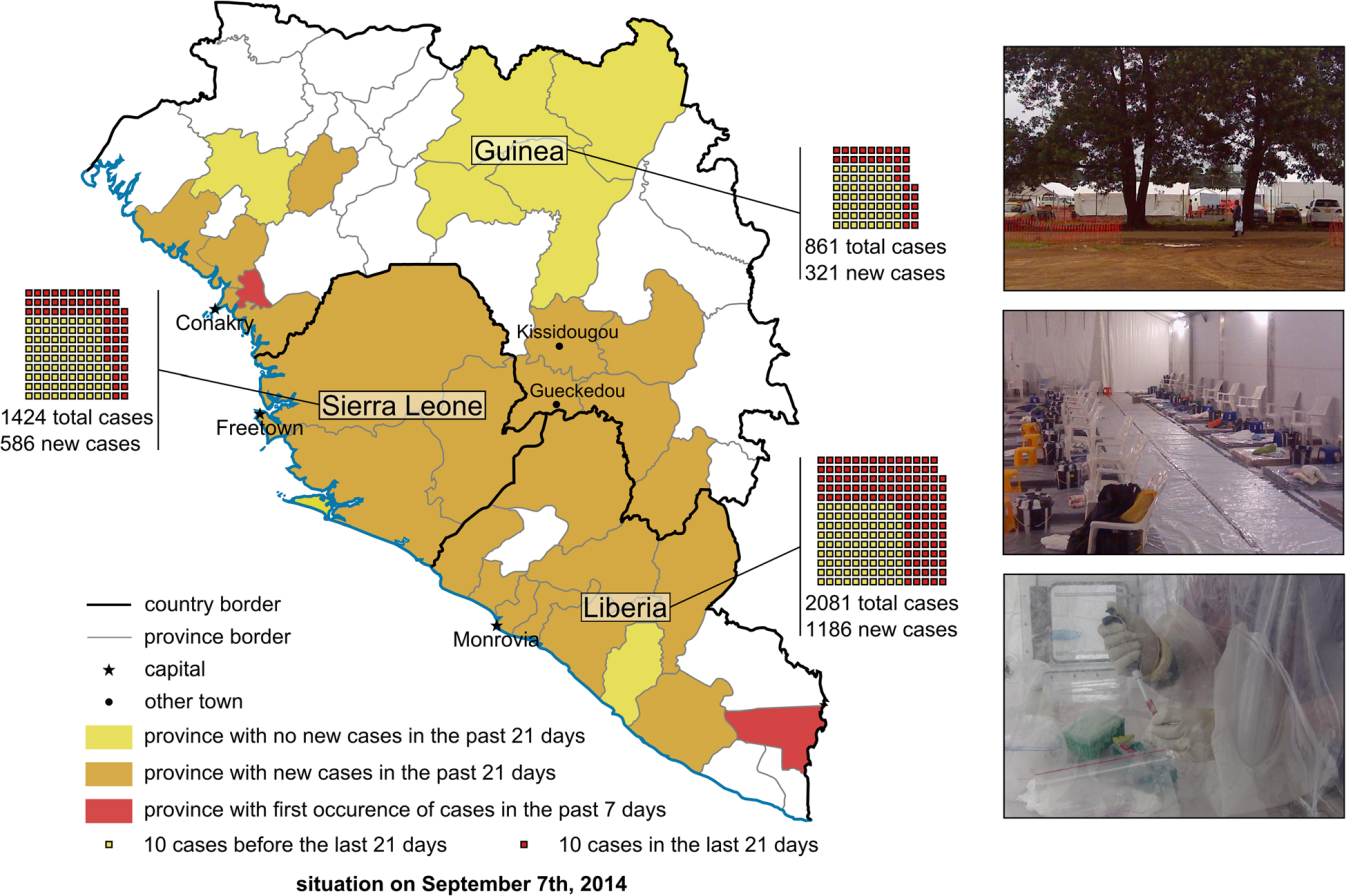
**Map of the West African ebolavirus outbreak as of 7 September 2014.** Country and province borders according to the CIA World Factbook are indicated as black or grey lines, respectively. Provinces with no new cases in the 21 days prior to 7^th^ September 2014 (according to the WHO situation report from 12^th^ September 2014) are highlighted in yellow, provinces with new cases in the 21 days prior to 7^th^ September in orange, and provinces showing cases for the first time in the 7 days prior to 7^th^ September are highlighted in red. Case numbers for each country are indicated, with each square representing 10 cases. Only countries with extensive person to person transmission are shown; Senegal and Nigeria are not shown. Inserts show a typical treatment and isolation facility set up as part of the international response to the outbreak, with the top insert showing the ELWA III treatment and isolation site, the middle insert showing the inside of a treatment tent, and the bottom insert showing a lab worker inactivating patient samples in a portable isolation chamber (pictures courtesy of Barry Fields (CDC Nairobi) and Dave Safronetz (NIH)).

The ongoing outbreak in West Africa of ebolavirus hemorrhagic fever (EHF) [1], lately also referred to as Ebola virus disease (EVD), has led to a surge in public interest and concern regarding this virus, which was first discovered in 1976 during simultaneous outbreaks in Zaire (now the Democratic Republic of the Congo) and Sudan [2]. Humans initially contract the virus either through contact with the infected reservoir, which is thought to be fruit bats, or by hunting and butchering of infected wildlife, particularly great apes. Since their discovery, ebolaviruses have caused frequent outbreaks almost exclusively in Central Africa. However, the recent emergence of *Zaire ebolavirus* in West Africa, resulting in what is the largest outbreak to date (Figure 1), with 4,390 cases and 2,226 deaths as of 7 September 2014, shows that ebolaviruses are more widely distributed than previously thought. While EHF is commonly associated with high case fatality rates (up to 90% for *Zaire ebolavirus*, approximately 50% for *Sudan ebolavirus*, and approximately 35% for *Bundibugyo ebolavirus*), the pathogenicity of *Taï Forest ebolavirus*, which was discovered in the mid-1990s in Ivory Coast, is unknown because only a single case has been reported, and *Reston ebolavirus*, which is found in the Philippines, is considered apathogenic for humans. Outbreaks are usually driven by human-to-human transmission as a result of direct contact with live or deceased patients and their body fluids, mainly during patient management and care, and participation in traditional local burial practices. Basic hygiene measures and barrier nursing techniques are usually sufficient to disrupt ebolavirus transmission and spread in the community. Nevertheless, because of its high case fatality rate and the absence of licensed vaccines or treatments, this virus is considered of the highest biosafety concern, restricting work on infectious virus to a few maximum containment laboratories worldwide. Despite the restricted and highly regulated handling of the pathogen, there have been considerable scientific achievements over the past years; however, many challenges remain in the public health sector in relation to identifying and managing cases and interrupting virus spread.

## Detecting and containing outbreaks

The first major challenge lies in outbreak recognition, as exemplified by the current EHF outbreak in West Africa, which took almost three months to recognize and even longer to appreciate as a major public health concern [1]. Initial disease symptoms, which occur suddenly after an incubation period of up to 21 days, are rather non-specific and include fever, malaise, headaches, muscle pain, nausea, vomiting and diarrhea [2,3]. More characteristic symptoms, such as hemorrhagic manifestations including vomiting of blood, nosebleed, bloody or tarry stool, and bleeding from injection sites, as well as a characteristic rash, appear later in the disease course, and are only obvious in about half of all patients. Pathogenesis involves a combination of immune suppression, vascular dysfunction, coagulopathy, and the dysregulation of cytokine responses similar to systemic inflammatory response syndrome (SIRS), ultimately resulting in multi-organ failure and death [2,4]. Importantly, similar symptoms, particularly in the initial stages of disease, can also be seen in patients infected with malaria and typhoid fever, as well as many other endemic infectious diseases [2,3] with which EHF can be confused. For non-endemic countries the fear of importation is justified, as demonstrated by past importations of ebolavirus into South Africa and the closely related marburgvirus into the USA and the Netherlands, although these remain rare events. However, the likelihood of importations has increased in association with the current outbreak in West Africa, where four capital cities that have multiple international travel connections are now affected, and with the evacuation of confirmed cases among foreign aid workers. Nevertheless, it is very important to realize that in most non-endemic countries ongoing person-to-person transmission, as we currently see in West Africa, is extremely unlikely, due to better access to professional health care, higher standards of hospital hygiene, patient management and diagnosis, safer burial practices, and the current high level of awareness among health care providers. Past experience has shown that even when diagnosis was delayed, secondary infections have not occurred during the rare incidences of imported infections, further indicating the critical importance of basic personal protection and hospital hygiene as key measures to control ebolavirus transmission.

Once an EHF outbreak is confirmed, rapid diagnosis based on quantitative real-time polymerase chain reaction (qRT-PCR) methodology, as well as serology and antigen detection, is available. However, given the remote locations in which outbreaks usually occur, this either involves time-consuming shipping of samples to more centrally located reference laboratories, or dispatching of mobile laboratories directly into the outbreak area [5]. The main strategy for outbreak management focuses on the reduction of secondary transmission by isolating infected individuals, the implementation of safe burial practices, contact tracing to disrupt infection chains, and education of the local population regarding risk reduction [6]. From a public health perspective, this strategy is paramount, and it has been successful in the past in controlling ebolavirus outbreaks; but it has so far had only limited effect on the current outbreak in West Africa. In terms of the individual patient, at this time, care is limited to supportive treatment to maintain vital function. The importance of providing the best possible care to patients is not only in helping to reduce case fatality rates, but also because it increases compliance with isolation procedures and, therefore, contributes to overall outbreak control.

To address the need for more rapid recognition of future EHF outbreaks, awareness among the medical community about EHF needs to remain high between outbreaks. Once cases are identified, a multidisciplinary approach, including the open and timely sharing of all relevant information, is imperative for a successful outbreak response.

## Developing and deploying countermeasures

**Figure 2:**
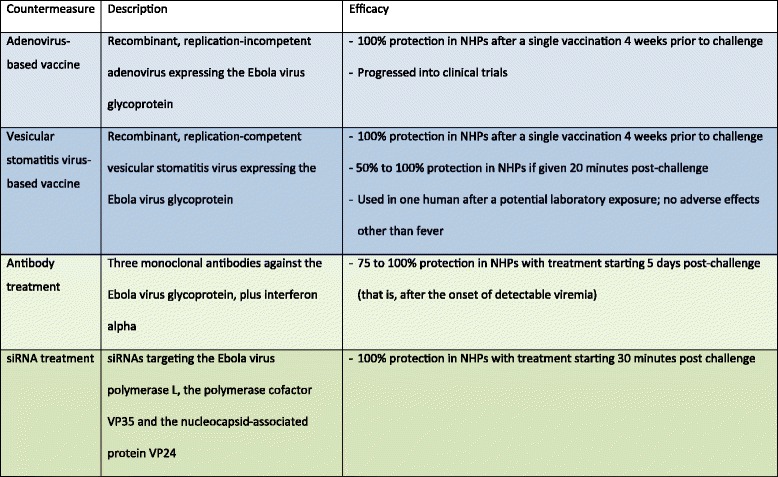
**Promising countermeasures against ebolaviruses.** Listed are the most advanced countermeasures (based on the authors’ judgment), all of which show 100% protection in non-human primates (NHPs). Vaccines are shown in blue and antivirals are shown in green. In the case of vaccines, only those that require a single vaccination are shown.

Currently no licensed vaccines or therapeutics are available for ebolaviruses. Over the past decade, however, funding has been made available for research into such countermeasures, resulting in encouraging progress on the preclinical level (Figure 2) [7]. Further, reverse genetics technologies have generated non-infectious systems that allow modeling of the complete ebolavirus life cycle without the need for maximum containment laboratories. Together with reporter-expressing recombinant ebolaviruses, these systems have significantly improved our ability to identify new antivirals [8]. The most promising antivirals at this point include the combinations of three monoclonal antibodies, which were protective in non-human primates (the most stringent disease model) up to two days after challenge, and when used in combination with interferon alpha were even protective if treatment was initiated three days after challenge, that is, after the onset of symptoms [9,10]. This approach is now further being developed as ZMapp, an improved antibody cocktail that is able to protect non-human primates with treatment starting as late as five days after challenge [11], and was recently administered to a small number of infected aid workers. Another very promising approach is the use of small interfering RNAs (siRNAs), which was also protective in non-human primates if given after exposure [12]. This approach is currently being developed as a commercial drug called TKM-Ebola, and while a phase I clinical trial was put on hold by the FDA in July 2014, on 7 August 2014 this ban was partially lifted, enabling the potential use of this drug in EHF patients.

Similar progress has been made with vaccines, with several platforms being highly protective in nonhuman primate models [13]. The furthest developed of these vaccines are based on viral vectors, since earlier attempts using inactivated vaccines were unsuccessful, and live-attenuated vaccines are generally considered too dangerous in the case of ebolaviruses. Viral vector-based vaccines are recombinant vaccines in which genes encoding proteins of a pathogenic virus such as ebolavirus are inserted into the genome of another virus that causes mild or no disease. The viruses mostly used in ebolavirus vaccines are either recombinant, replication-deficient adenoviruses (Ads) or attenuated vesicular stomatitis viruses (VSVs), both of which are not known to cause serious side effects in humans. The ebolavirus genes encode proteins that can be recognized by the immune system but do not cause disease. The adenovirus platform uses a non-replicating recombinant adenovirus carrying the genetic information for the ebolavirus surface glycoprotein (GP) or for both GP and the ebolavirus nucleoprotein. Vaccination with this virus leads to the production of these proteins in the vaccinated individual, resulting in the development of an adaptive immune response. Most studies were performed using the human Ad5 serotype, with which many people have been infected at some point in their life. While in non-human primate studies this vaccine confers complete protection if given four weeks prior to challenge, in humans there are significant problems with preexisting immunity against the Ad5 serotype, so that individuals who have been previously exposed to Ad5 fail to mount an immune response to the ebolavirus component of the vaccine. While the Ad5-based vaccine was safe and immunogenic in a phase I clinical trial, the immune response was likely insufficient to confer protection [14]. The problem of preexisting immunity has been addressed by using different serotypes, including the rare human serotypes Ad26 and Ad35, chimpanzee Ad3, Ad7, and Ad63, as well as simian Ad21. Limited studies showed 100% protective efficacy in non-human primates after a single vaccination for the chimpanzee Ad3-based vaccine, although a booster immunization was required to achieve long-lasting immunity [15]. A phase I clinical trial with this vaccine began in early September 2014.

The VSV platform uses an attenuated replication-competent recombinant VSV that contains the ebolavirus GP gene instead of its own glycoprotein gene, leading to the production of virus particles that incorporate the ebolavirus glycoprotein into the virus envelope, as well as the production of this protein in vaccinated individuals. In extensive studies this virus has been shown to protect 100% of non-human primates if given either three or four weeks prior to challenge [16], and surprisingly it also showed some potential in post-exposure use, with 50 to 100% survival of non-human primates if the vaccine was administered 20 to 30 minutes after challenge, depending on the species of the challenge ebolavirus. This vaccine platform has not progressed into clinical trials yet, but it was shown to be safe in severely immunocompromised non-human primates, and also did not cause neural pathology even after intrathalamic administration to non-human primates [17,18]. This is important because although VSV is non-pathogenic in humans, it can be neurovirulent in mice, and the possibility of neural pathology in humans must be ruled out - especially since, in contrast to the adenovirus-based vaccine, the VSV-based vaccine remains replication-competent. There has been a single use of this vaccine as a potential post-exposure treatment after a needle-stick incident in a laboratory worker in Hamburg, Germany, with no side effects other than a transient fever [19]. A first phase I clinical trial is planned for fall 2014.

One pressing and extremely difficult question is whether such experimental treatments and vaccines should be used in the current outbreak. The recent WHO declaration to ethically approve the use of experimental drugs and vaccines under certain circumstances is likely to improve current outbreak response strategies. However, one needs to remain realistic regarding what can be done during the current outbreak, given both the extremely limited amount of clinical grade material that is available and the lack of human safety data for any of the promising experimental drugs and vaccines. Clearly, improving the chances of survival of an infected patient is highly desirable. Improved survival might also help to change a perception in the population that the isolation wards are ‘death traps’ rather than medical care facilities, which could lead in turn to improved compliance with conventional outbreak control measures. However, because of the general lack of human safety and efficacy data for these experimental drugs and vaccines there is a risk of adverse effects and/or ineffectiveness, which could result in the perception that developed countries are experimenting on African patients. The consequent deterioration in the relationship between the affected African population and foreign health care workers might decrease compliance with outbreak control measures and may even lead to aggression, ultimately resulting in further or even complete loss of outbreak control. This risk scenario applies in particular to the more recent demands for the testing of certain drugs approved/licensed for other medical applications, which counteract human host responses that have been implied in the pathogenesis of EHF, but without preclinical efficacy data for those drugs against EHF. Safety of a drug targeting a host response mechanism predicted to be relevant during ebolavirus infection seems too weak as a justification considering the potentially disastrous consequences of a failure in efficacy on the ground.

Implementation of any countermeasure needs a well designed strategy, and this is particularly the case when supplies are limited, as they are at the moment. In addition, different situations may call for the use of different countermeasures. For example, foreign health care workers may be best served by using a safe and effective vaccine approach, whereas confirmed patients will need a therapeutic approach such as an antiviral (for example ZMapp or TKM-Ebola). Local health care staff and family members may benefit from a ring vaccination approach (that is, vaccination of actual or potential contacts to infected individuals) using a fast-acting vaccine such as the recombinant VSV, or prophylactic treatment using an antiviral or therapeutic. If experimental countermeasures are used, clinical safety and efficacy data should certainly be collected whenever possible. The question, however, is how that can be integrated with currently ongoing outbreak control measures, and whether we can ethically justify control groups if the countermeasures have high prediction values for success. In addition, giving some patients and health care or aid workers priority for treatment or vaccination will certainly create discord among the affected population, aid organizations, and governments, as decisions are unlikely to be seen as politically, ethnically and ethically correct by all parties. Regardless, targeting of health care workers with vaccination might be one justified instance for the following reasons: first, they are at significant risk for acquiring EFH as an inherent part of their work; second, they are absolutely essential to the on-going management of the outbreak, and thus for public health; and third, they might be in a better position to give informed consent to receive an experimental countermeasure. Despite all of the complications, we should not forget that using countermeasures could have a tremendous positive impact on the current outbreak, and provide us with a great chance to gain experiences that can then be applied during the inevitable future outbreaks.

The decisions regarding whether to deploy experimental countermeasures, which countermeasure to deploy, and how to do so are extremely complex and difficult, and will have to involve a careful risk/benefit evaluation, not only on the level of an individual patient, but also for overall outbreak management. These decisions should be made jointly with all affected parties, including scientists and public health experts, the aid organizations involved in outbreak management, and most importantly representatives of the people directly affected by the outbreak. As a note of caution, any use of countermeasures should not affect strengthening the traditional public health response measures, which have a very successful track record and are likely to be successful in this outbreak if widely and rigorously applied.

## Future directions

Research has made significant progress in combating ebolavirus infections. We now have to translate these scientific advances into tangible benefits for the people affected by this devastating disease. Events in the current outbreak are rapidly developing, and it is impossible for the authors to predict what measures will have been taken by the time this article is published. However, regardless of whether experimental countermeasures are eventually used in the current ebolavirus outbreak, it is clear that their use can only be considered a last resort and that the strengthening of traditional public health and outbreak response measures are of paramount importance. For the future, the major challenge lies in advancing the experimental treatments and vaccines towards licensing for human use. Further, deployment strategies have to be put into place both for delivering countermeasures into an outbreak area, and for their administration. It will also be necessary to consider the possibility that infections will be imported into countries in which infections with ebolaviruses do not normally occur, and to develop management plans. Again, overcoming this challenge will require collaboration between scientists, aid organizations, pharmaceutical companies, local communities, regulatory bodies, and governments. Noteworthy is the currently increasing interest in industry as well as in politics in ebolaviruses, something that has to be seen as a positive development [20]. Finally, future efforts should not be restricted to ebolaviruses, but also include other communicable pathogens with the potential to cause devastating outbreaks, such as the closely related marburgviruses. Even once the current EHF outbreak in West Africa ceases, it is only a matter of time until the next outbreak strikes. One should wisely use this opportunity to be proactive.
